# Supplementary material for: Functional data analysis characterizes the shapes of the first COVID-19 epidemic wave in Italy
Source: Sci Rep. 2021 Aug 30;11:17054. doi: 10.1038/s41598-021-95866-y (PMC8405612; doi:10.1038/s41598-021-95866-y)
Supplement: Supplementary file 1 — Supplementary Information. [file 41598_2021_95866_MOESM1_ESM.pdf]

Functional Data Analysis characterizes the shapes of the first COVID-19 epidemic wave in Italy
SUPPLEMENTAL MATERIAL

Tobia Boschi[1], **Jacopo Di Iorio**[2], **Lorenzo Testa**[2], **Marzia A. Cremona**[1,3,4,*], **and Francesca Chiaromonte**[1,2,*]

[1]Penn State University, Dept. of Statistics and Huck Institutes of the Life Sciences, University Park, PA 16802, USA
[2]Sant'Anna School of Advanced Studies, Institute of Economics and EMbeDS, Pisa, 56127, Italy
[3]Université Laval, Dept. of Operations and Decision Systems, Québec, G1V 0A6, Canada
[4]CHU de Québec – Université Laval Research Center, Québec, G1V 4G2, Canada
[*]co-corresponding authors; marzia.cremona@fsa.ulaval.ca, fxc11@psu.edu

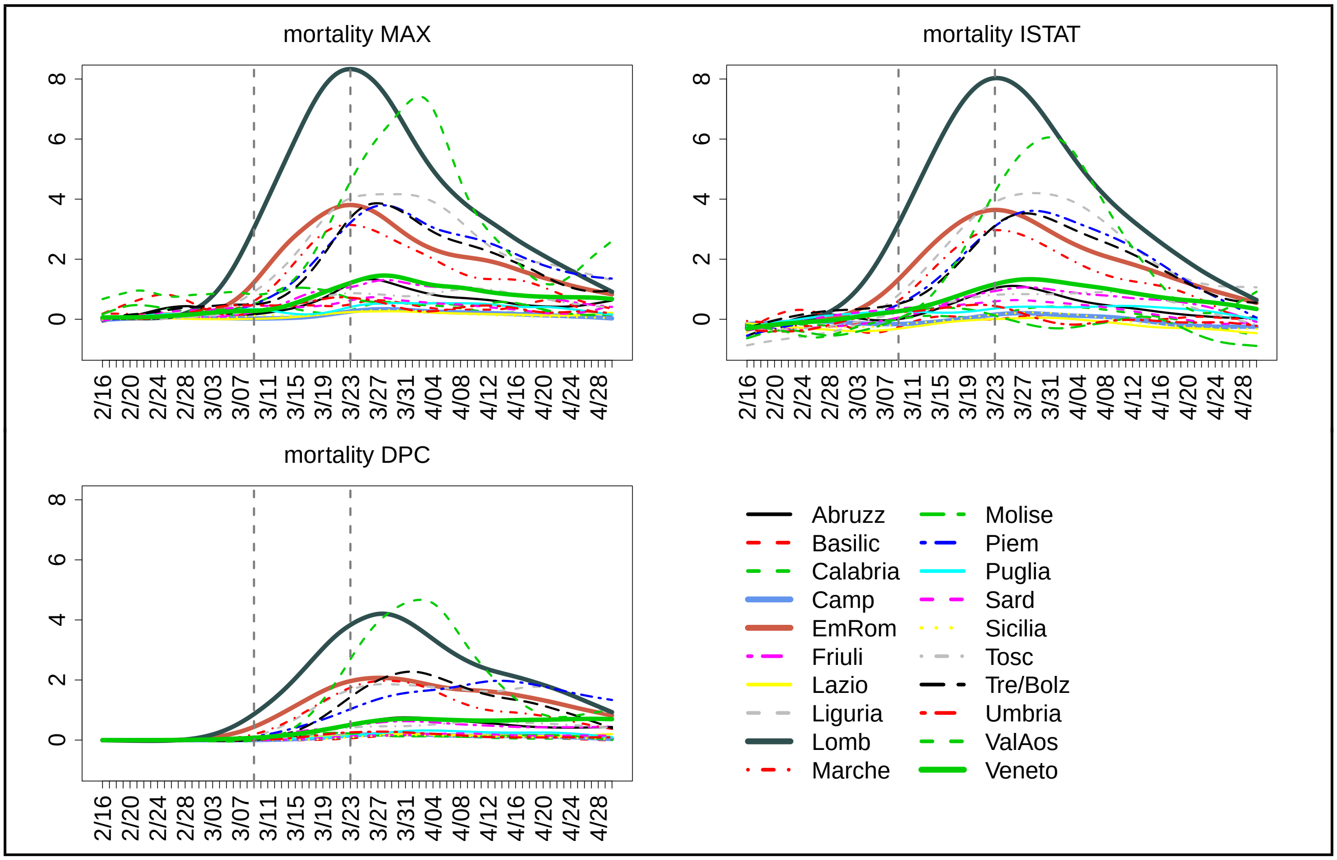

Figure S1. Unshifted mortality curves. MAX, ISTAT and DPC mortality curves (per 100,000 inhabitants) without shift. Vertical lines show the days corresponding to the national lock down (March 9) and the suspension of all non-essential production activities (March 23).

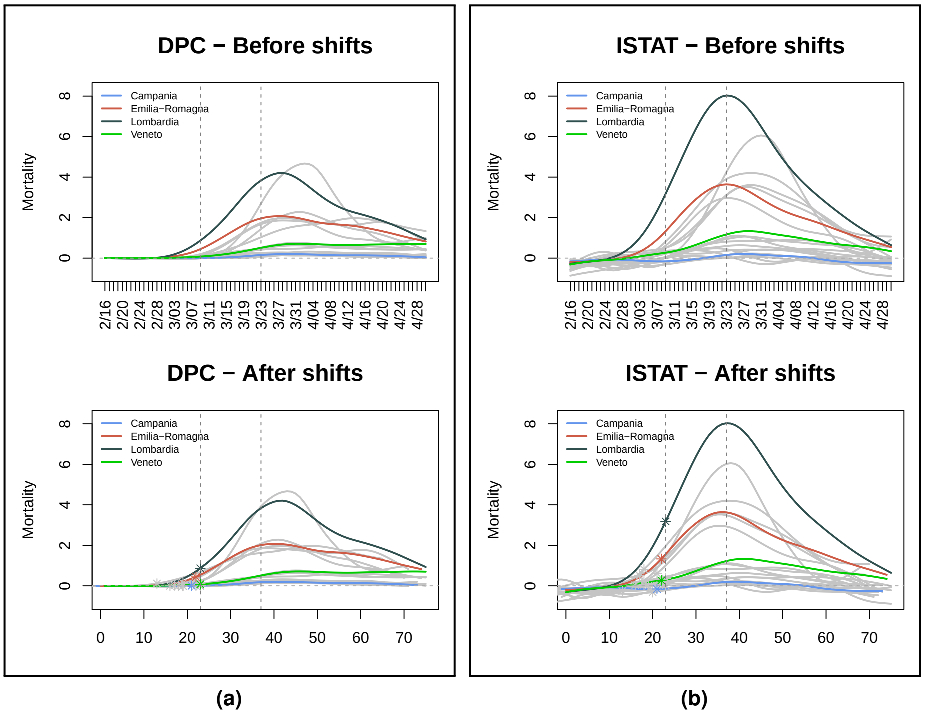

Figure S2. Mortality curves. **(a)**: DPC mortality curves (per 100,000 inhabitants) in the 20 Italian regions – before (top) and after (bottom) the shifts produced by *probKMA* run with $K = 2$. **(b)**: ISTAT mortality curves (per 100,000 inhabitants) in the 20 Italian regions – before (top) and after (bottom) the shifts produced by *probKMA* run with $K = 2$. In all panels, vertical lines mark the dates of the national lock-down (March 9) and the suspension of all non-essential production activities (March 23). In the bottom panels, vertical lines still show these dates without shifts, stars on the curves mark the lock-down after the region-specific shifts.

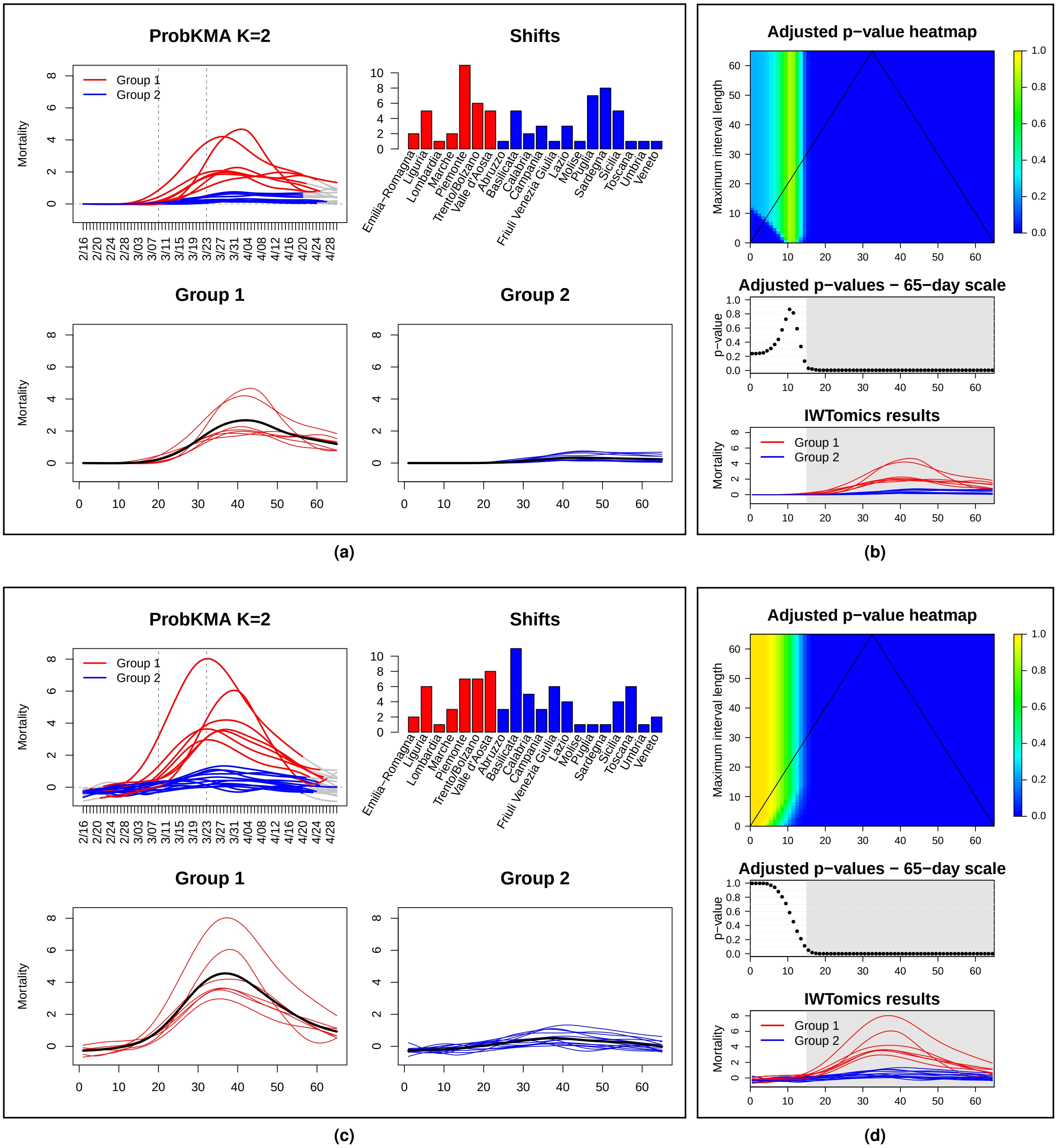

Figure S3. Characterizing two epidemics. Results of *probKMA* and *IWTomics* on **(a)**-**(b)** DPC mortality curves and **(c)**-**(d)** ISTAT curves. **(a)** and **(c)**: Mortality curves are shown in the top left panel with portions identified by *probKMA* with $K = 2$ in red (Group 1; "exponential" pattern) and blue (Group 2; "flat(tened)" pattern). The curve portions are shown again, this time aligned with each other and separated by group, in the bottom panels. Black lines indicate group averages. The shifts produced by *probKMA* are shown in the top right panel. **(b)** and **(d)**: Shifted Group 1 and Group 2 mortality curves are tested against each other with *IWTomics*. The heatmap at the top shows p-values adjusted at all possible scales (from 1 to 65 days). The middle panel shows in detail the top-most row of the heatmap; i.e. the p-values adjusted across the whole 65-day interval (the 65 different values are reported in Table S1). The bottom panel shows again the shifted curves. Gray areas in the middle and bottom panels mark days when the difference between the two groups is significant (adjusted p-value $< 5\%$).

Table S1. ***IWTomics*** **adjusted p-value curves.** Values of the IWTomics adjusted p-values for each time point and each of the three dataset. Note that the smallest possible p-value is 0.001 since we are employing 1000 permutations in the IWTomics test.

	MAX	ISTAT	DPC
1	0.648	0.996	0.238
2	0.651	0.998	0.240
3	0.648	0.998	0.242
4	0.634	0.996	0.252
5	0.625	0.988	0.275
6	0.608	0.967	0.308
7	0.581	0.939	0.364
8	0.538	0.878	0.442
9	0.513	0.808	0.575
10	0.462	0.711	0.723
11	0.420	0.583	0.864
12	0.381	0.454	0.813
13	0.335	0.319	0.588
14	0.275	0.211	0.337
15	0.210	0.114	0.130
16	0.143	0.048	0.032
17	0.073	0.019	0.018
18	0.022	0.004	0.008
19	0.001	0.002	0.003
20	0.001	0.001	0.002
21	0.001	0.001	0.001
22	0.001	0.001	0.001
23	0.001	0.001	0.001
24	0.001	0.001	0.001
25	0.001	0.001	0.001
26	0.001	0.001	0.001
27	0.001	0.001	0.001
28	0.001	0.001	0.001
29	0.001	0.001	0.001
30	0.001	0.001	0.001
31	0.001	0.001	0.001
32	0.001	0.001	0.001
33	0.001	0.001	0.001
34	0.001	0.001	0.001
35	0.001	0.001	0.001
36	0.001	0.001	0.001
37	0.001	0.001	0.001
38	0.001	0.001	0.001
39	0.001	0.001	0.001
40	0.001	0.001	0.001
41	0.001	0.001	0.001
42	0.001	0.001	0.001
43	0.001	0.001	0.001
44	0.001	0.001	0.001
45	0.001	0.001	0.001
46	0.001	0.001	0.001
47	0.001	0.001	0.001
48	0.001	0.001	0.001
49	0.001	0.001	0.001
50	0.001	0.001	0.001
51	0.001	0.001	0.001
52	0.001	0.001	0.001
53	0.001	0.001	0.001
54	0.001	0.001	0.001
55	0.001	0.001	0.001
56	0.001	0.001	0.001
57	0.001	0.001	0.001
58	0.001	0.001	0.001
59	0.001	0.001	0.001
60	0.001	0.001	0.001
61	0.001	0.001	0.001
62	0.001	0.001	0.001
63	0.001	0.001	0.001
64	0.001	0.001	0.001
65	0.001	0.001	0.001

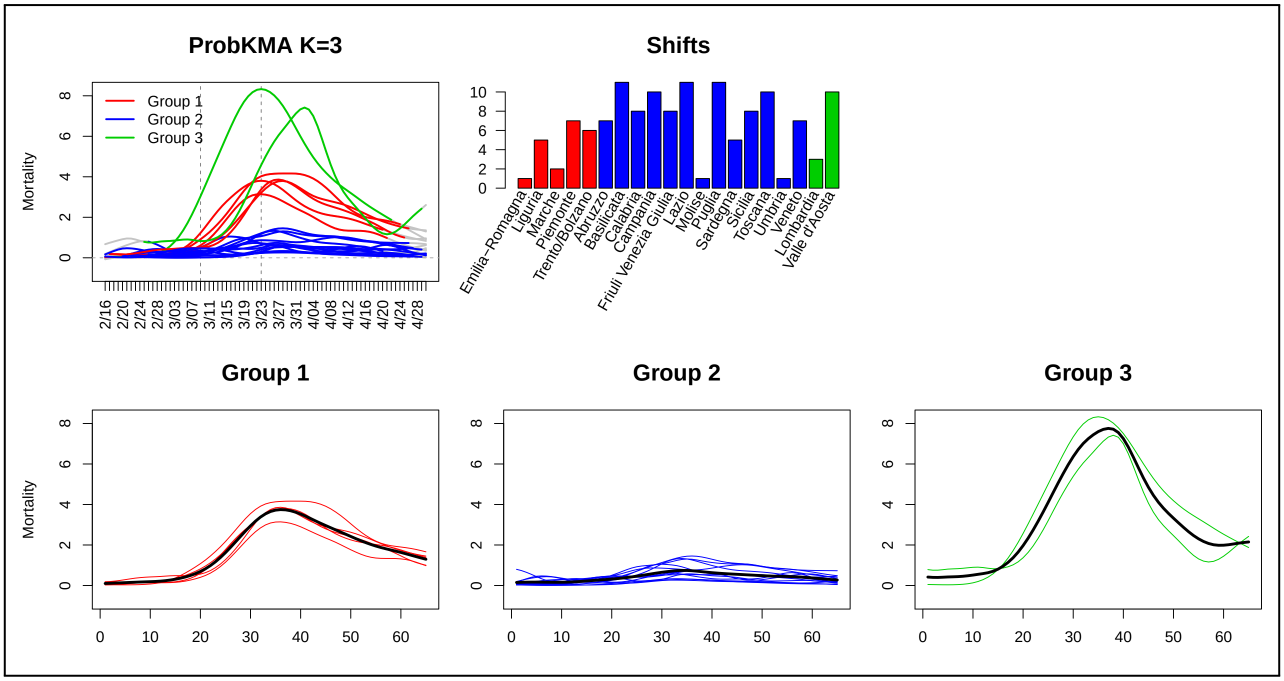

ProbKMA K=3
Group 1
Group 2
Group 3
Mortality
2/16
2/20
2/24
2/28
3/03
3/07
3/11
3/15
3/19
3/23
3/27
3/31
4/04
4/08
4/12
4/16
4/20
4/24
4/28
Shifts
Emilia−Romagna
Liguria
Marche
Piemonte
Trento/Bolzano
Abruzzo
Basilicata
Calabria
Campania
Friuli Venezia Giulia
Lazio
Molise
Puglia
Sardegna
Sicilia
Toscana
Umbria
Veneto
Lombardia
Valle d'Aosta
Group 1
Group 2
Group 3

(a)

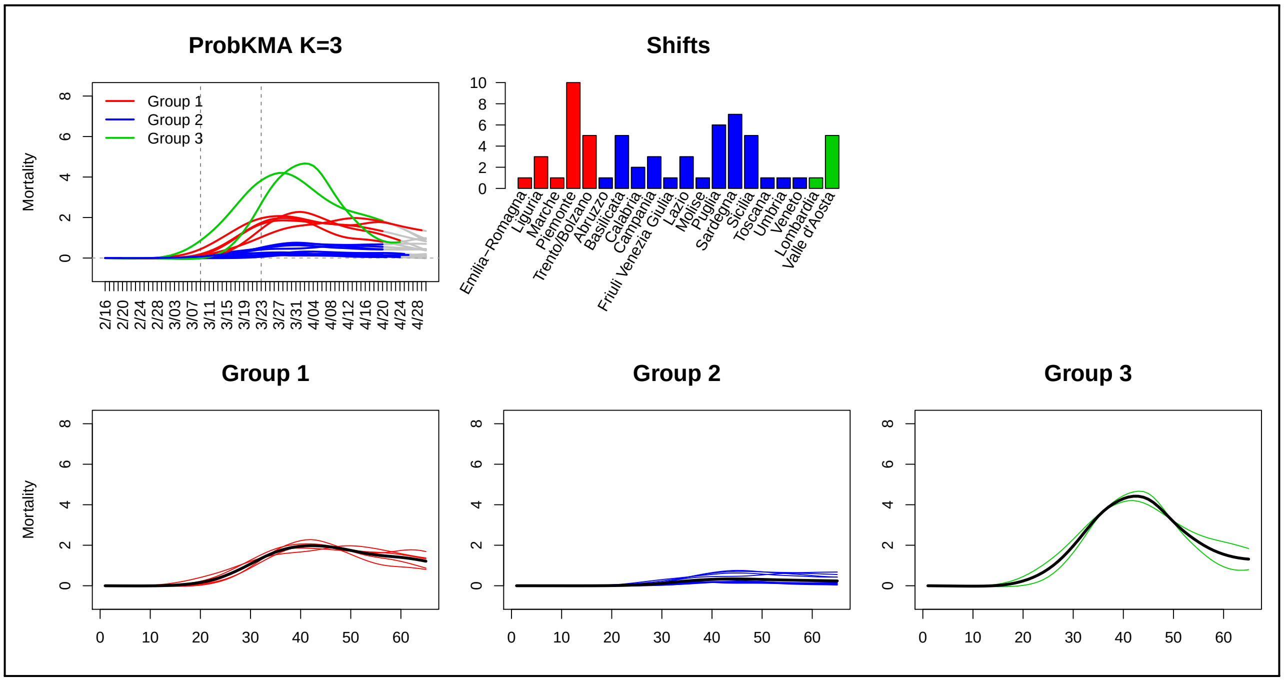

ProbKMA K=3
Group 1
Group 2
Group 3
Mortality
2/16
2/20
2/24
2/28
3/03
3/07
3/11
3/15
3/19
3/23
3/27
3/31
4/04
4/08
4/12
4/16
4/20
4/24
4/28
Shifts
Emilia−Romagna
Liguria
Marche
Piemonte
Trento/Bolzano
Abruzzo
Basilicata
Calabria
Campania
Friuli Venezia Giulia
Lazio
Molise
Puglia
Sardegna
Sicilia
Toscana
Umbria
Veneto
Lombardia
Valle d'Aosta
Group 1
Group 2
Group 3

(b)

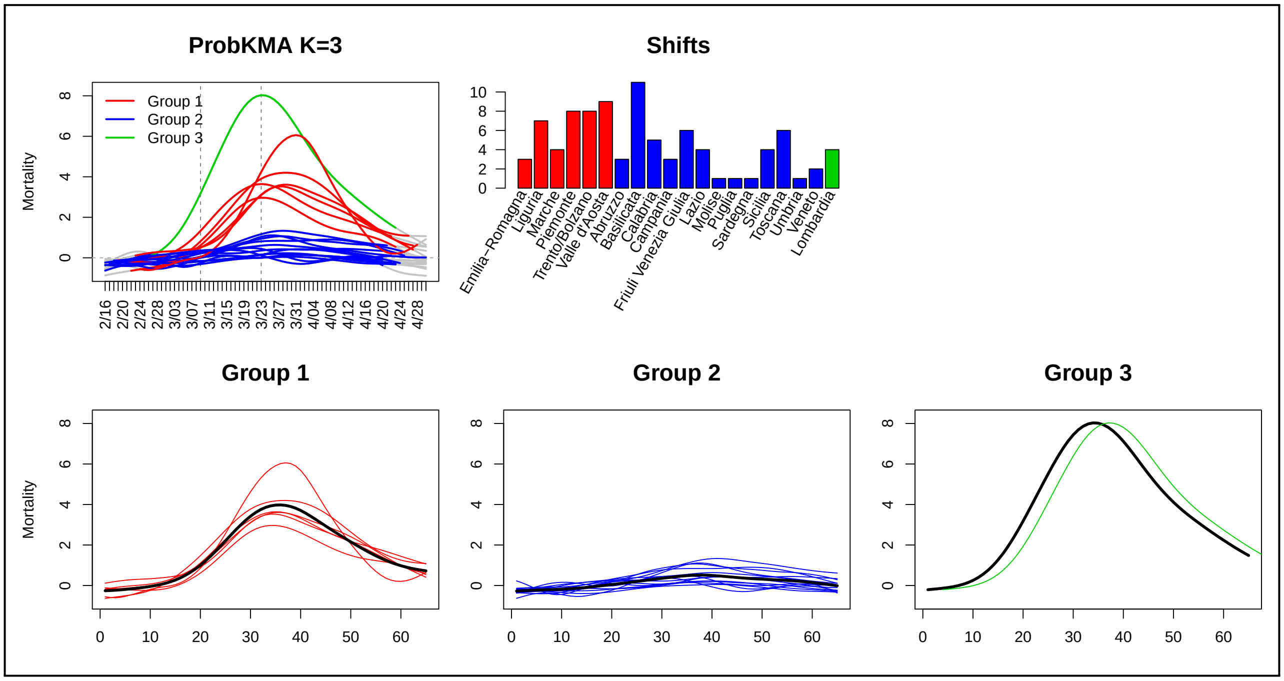

(c)

Figure S4. Characterizing three epidemics. Results of *probKMA* with $K = 3$ on **(a)** MAX mortality curves, **(b)** DPC mortality curves and **(c)** ISTAT curves. Mortality curves are shown in the top left panel with portions identified by *probKMA* with $K = 3$ in red (Group 1; "exponential" pattern), blue (Group 2; "flat(tened)" pattern) and green (Group 3; "extreme" pattern). The curve portions are shown again, this time aligned with each other and separated by group, in the bottom panels. Black lines indicate the average curves of the group. The shifts produced by *probKMA* are shown in the top right panel.

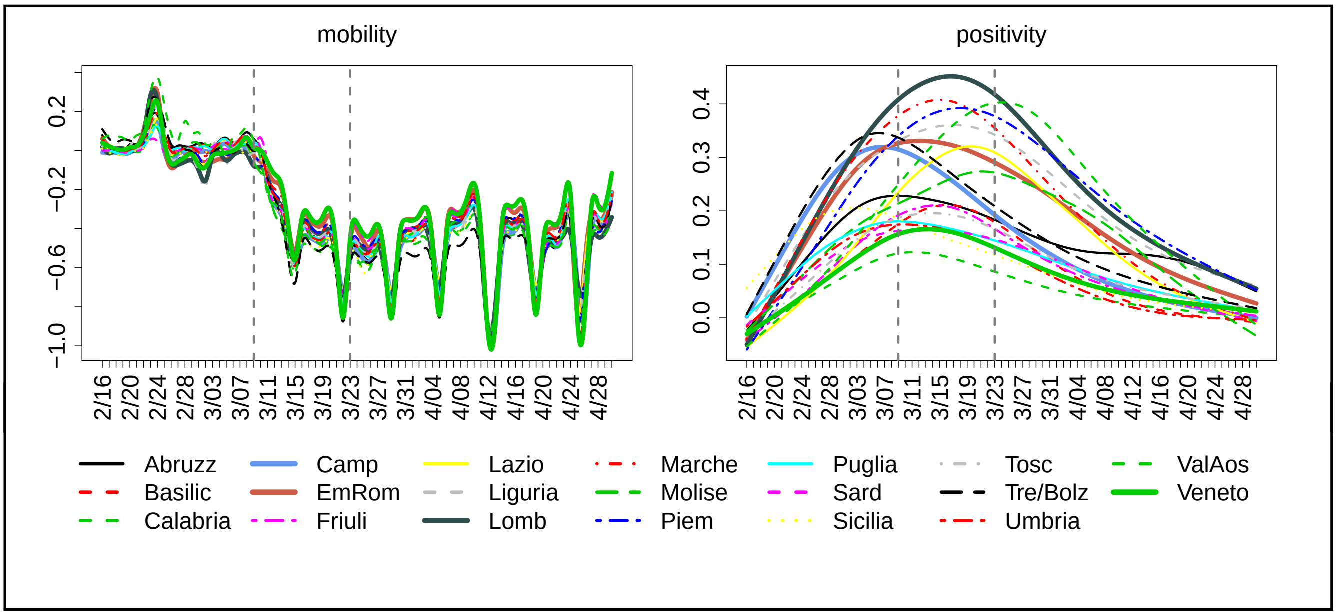

Figure S5. Unshifted mobility and positivity curves. Local mobility and positivity curves without shift in the 20 Italian regions. Vertical lines show the days corresponding to the national lock down (March 9) and the suspension of all non-essential production activities (March 23).

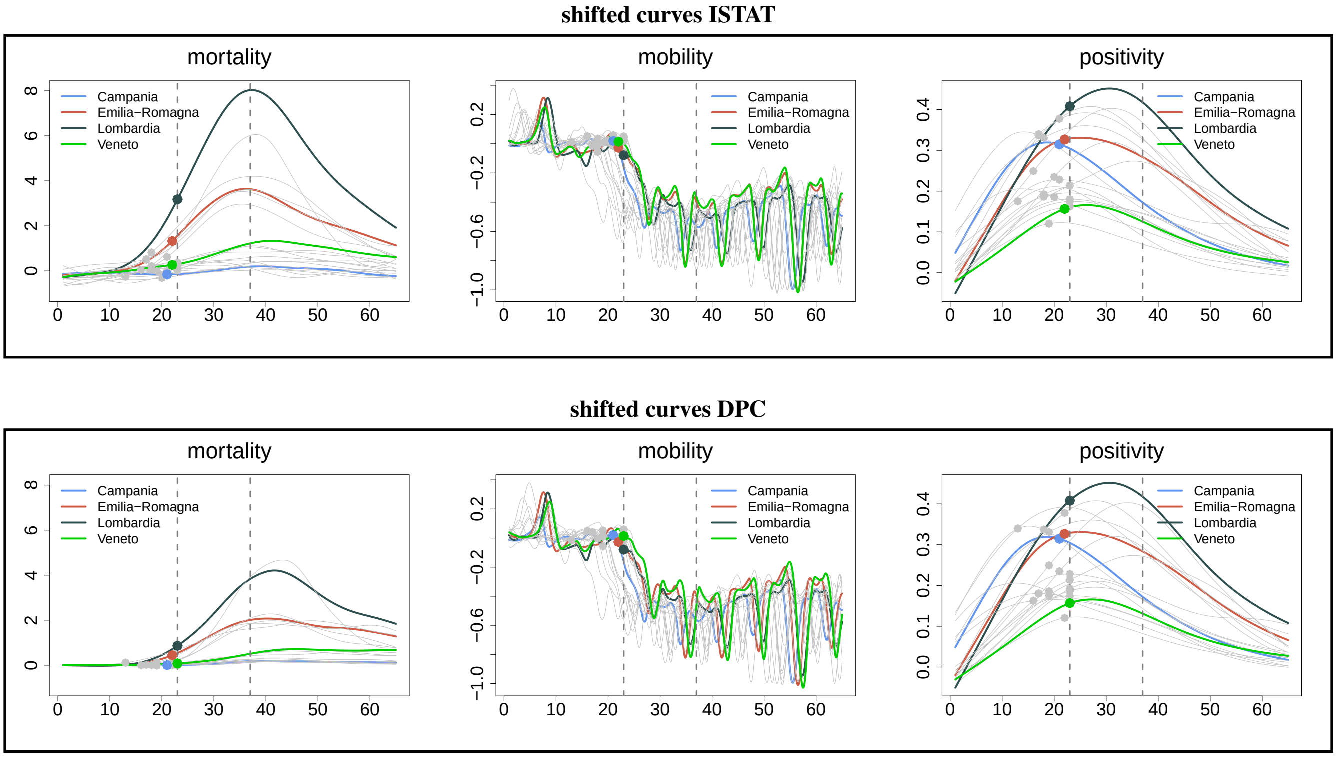

Figure S6. **Shifted curves for ISTAT and DPC.** Mortality (per 100,000 inhabitants), mobility, and positivity curves after the shifts produced by *probKMA* with K=2. Vertical lines mark the dates of the national lock-down (March 9) and the suspension of all non-essential production activities (March 23) without shifts. Stars on the curves mark the lock-down after the region-specific shifts.

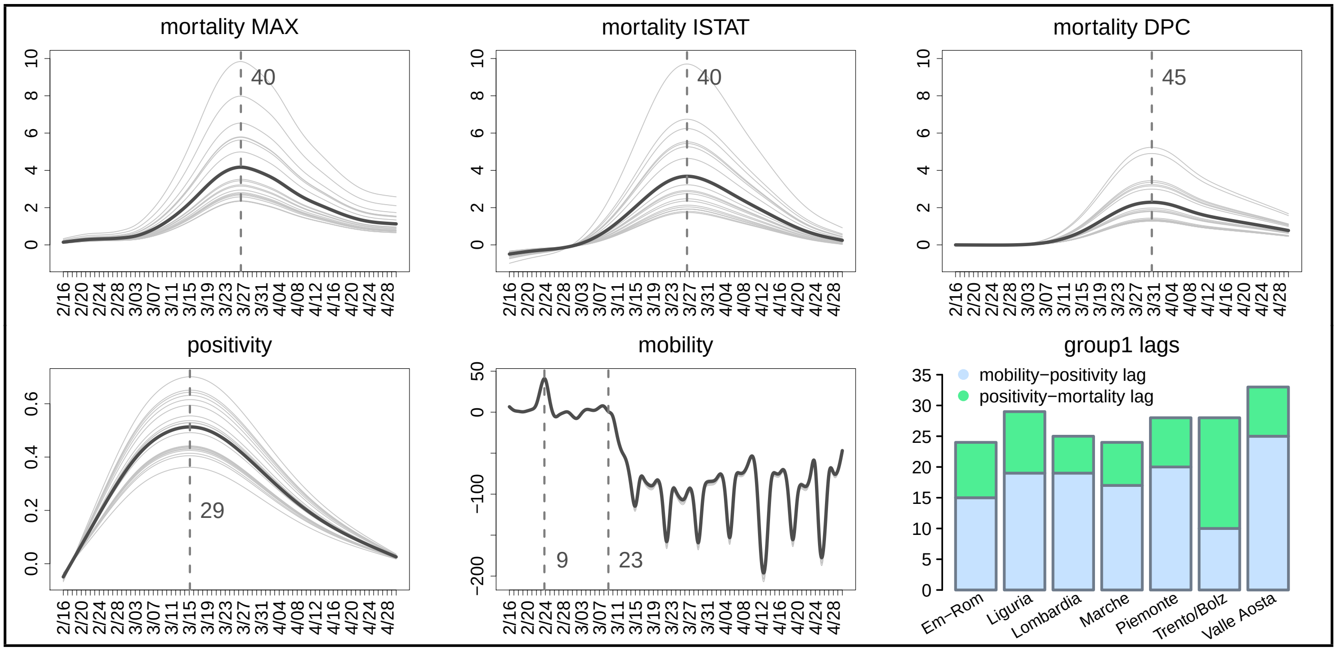

Figure S7. Lags characterization. Projections on the first Principal Components of mortality curves (MAX, ISTAT, and DPC – upper panels), positivity curves (lower-left panel) and mobility curves (lower-mid panel). Here the curves are not shifted, so the horizontal axis represents calendar dates. Projecting the curves on their first Principal Components produces an additional de-noising, which inherently aligns all the peaks. The thick dark lines are average curves in each panel, and the vertical lines mark the date of their maxima – also expressed in number of days from February 16th. For mobility, we also mark the beginning of the national lockdown (March 9th; 23 days from February 16th), which corresponds to a severe drop in the curves. We notice lags of about 20 days and 1 week between, respectively, the peak and the stark drop in mobility, and the peak in positivity (on average across all regions). The lag between the peak in positivity and the peak in mortality is about 10 days (again on average across all regions). The lower-right panel shows the individual mobility-positivity and positivity-mortality lags for each region in group 1 (those characterized by an “exponential" epidemic pattern). Albeit with some variation across regions, adding up these lags produces an overall delay of approximately 1 month between the peak mobility and that of mortality.

Table S2. Functional regression models (in-sample) R^2, LOO-CV R^2 and partial R^2s. For each functional linear model which regresses mortality on the covariates listed in the first column, the table reports the (in-sample) R^2, the LOO-CV R^2 and the partial R^2s.

	**MAX**			**ISTAT**			**DPC**		
**covariates**	R^2	LOO-CV R^2	partial R^2s	R^2	LOO-CV R^2	partial R^2s	R^2	LOO-CV R^2	partial R^2s
mob	0.79	0.54	-	0.63	0.47	-	0.62	0.33	-
pos	0.75	0.47	-	0.71	0.44	-	0.73	0.47	-
mob + pos	0.90	0.52	mob: 0.62<br>pos: 0.53<br>-	0.93	0.64	mob: 0.74<br>pos: 0.80<br>-	0.90	0.69	mob: 0.66<br>pos: 0.76<br>-
mob + pos + pc1	0.94	0.70	mob: 0.66<br>pos: 0.61<br>pc1: 0.39	0.93	0.62	mob: 0.57<br>pos: 0.67<br>pc1: 0.03	0.94	0.68	mob: 0.43<br>pos: 0.57<br>pc1: 0.00

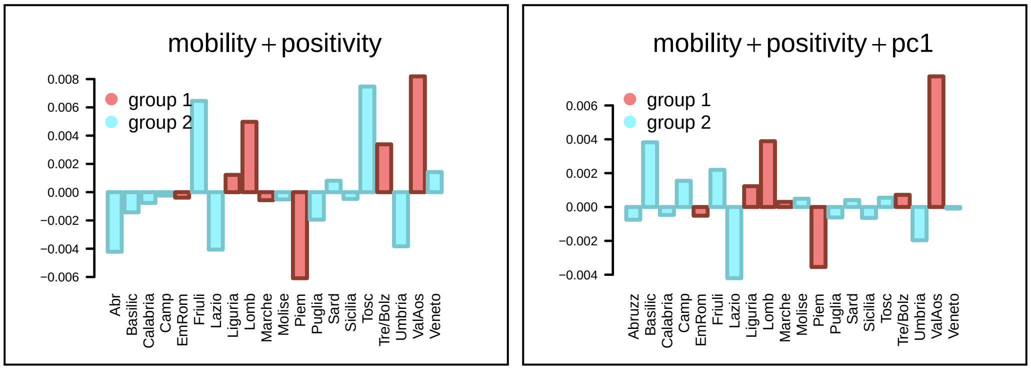

(a) (b)

Figure S8. MAX mortality residuals. **(a):** residuals of the function-on-function regression of MAX mortality on local mobility and positivity. **(b):** residuals of the function-on-function regression of MAX mortality on local mobility, positivity, and the first principal component of the top 5 covariates. In both panels curves from Group 1 are in red, and curves from Group 2 are in blue. Residuals with positive signs indicate regions for which the true mortality curve is above the estimated mortality curve. Conversely, residuals with negative signs indicate regions for which the true mortality curve is below the estimated mortality curve.

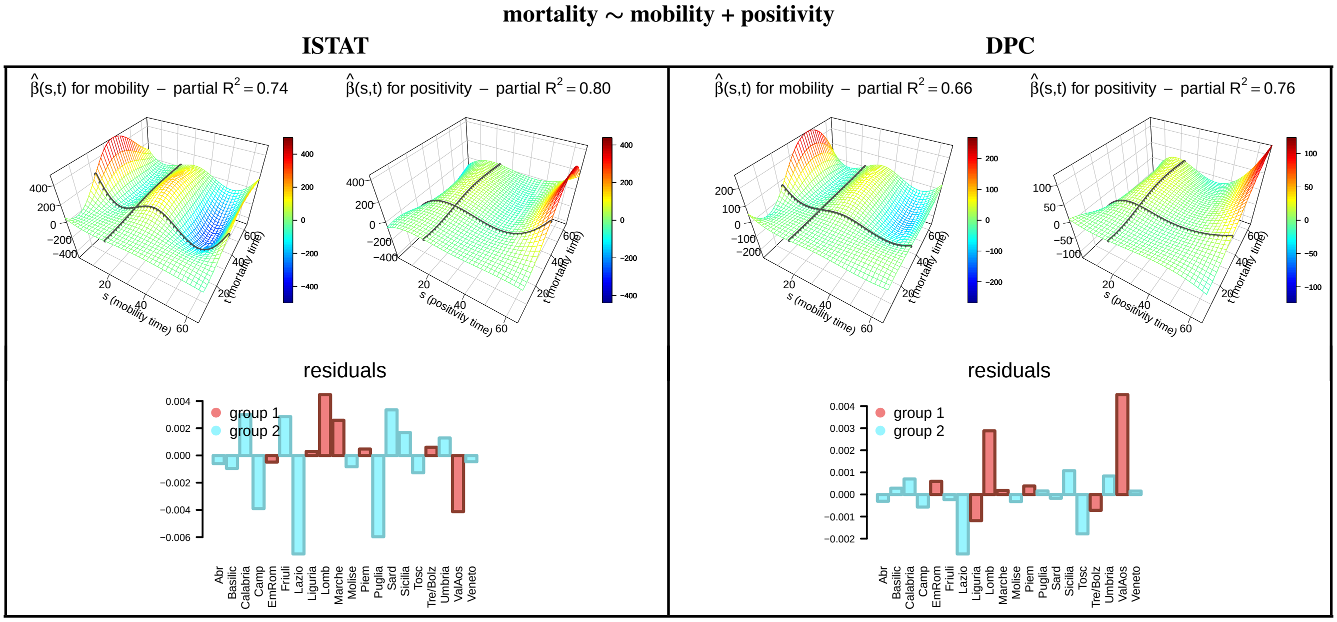

Figure S9. Associating mortality to mobility and positivity - ISTAT and DPC. Results from the joint function-on-function regression of ISTAT and DPC mortality on mobility and positivity. The top row shows the estimated effect surfaces (the March 9 date is marked) with respective partial R^2 (for in-sample R^2 and LOO-CV R^2 see Table S2). The bottom row shows the regression residuals (for barplots interpretation see Fig. S8)

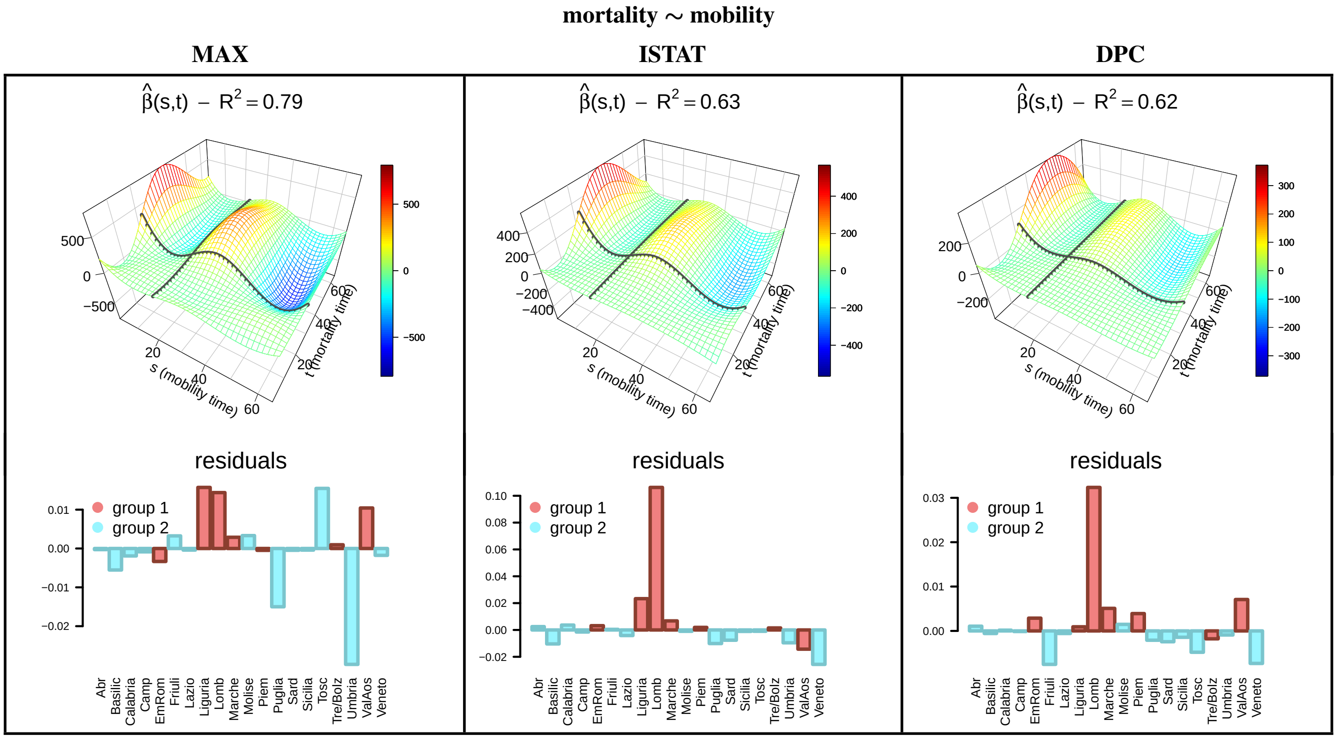

Figure S10. Associating mortality to mobility. Results from the function-on-function regression of mortality on local mobility. The top row displays the estimated effect surface (the March 9 date is marked) with respective in-sample R^2 (for LOO-CV R^2 see Table S2). The bottom row displays the regression residuals (for barplots interpretation see Fig. S8).

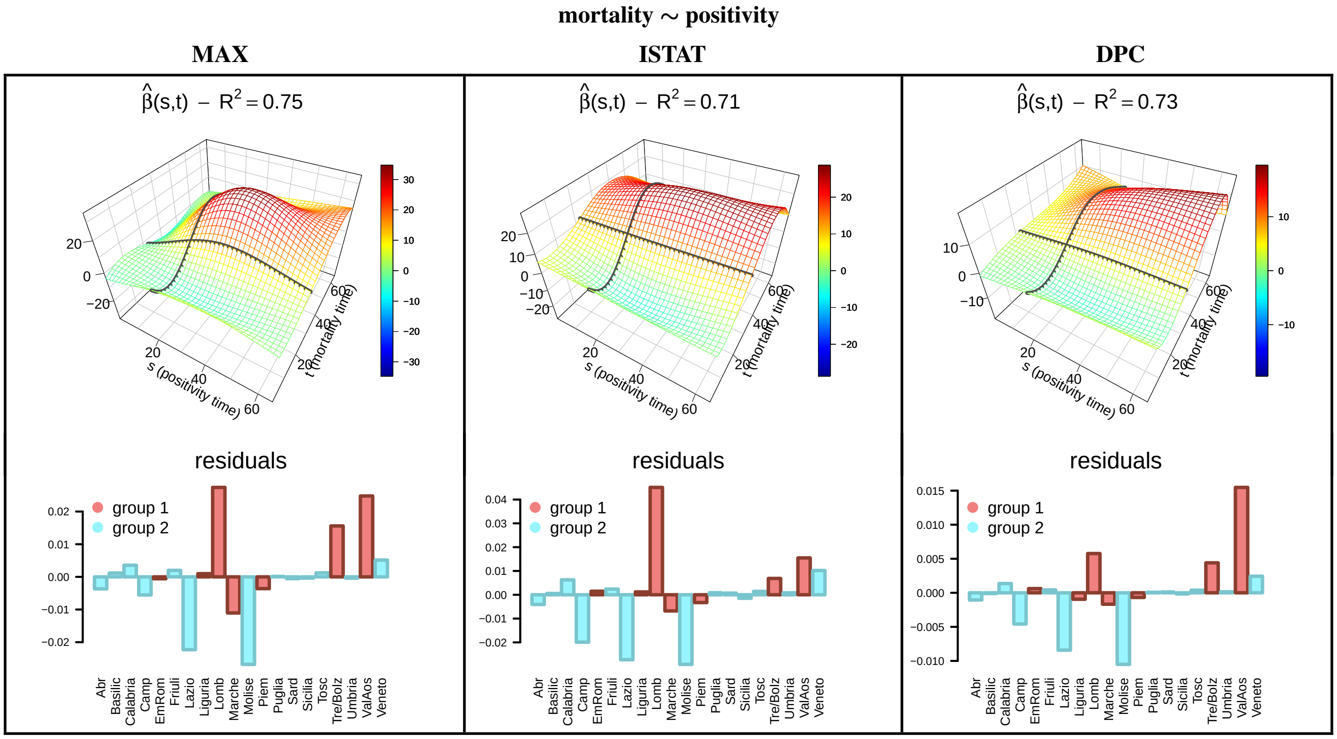

Figure S11. **Associating mortality to positivity.** Results from the function-on-function regression of mortality on positivity. The top row displays the estimated effect surface (the March 9 date is marked) with respective in-sample R^2 (for LOO-CV R^2 see Table S2). The bottom row displays the regression residuals (for barplots interpretation see Fig. S8).

Table S3. Covariates. List of all scalar covariates considered.

Covariate	Year and Source
Resident population, units	2018, ISTAT
Land area, hectares	2018, ISTAT
% population over 65	2018, ISTAT
% population over 70	2018, ISTAT
% population over 80	2018, ISTAT
% population over 85	2018, ISTAT
% male over 18	2018, ISTAT
% female over 18	2018, ISTAT
Employees in large supermarket chains, units	2018, ISTAT
Department stores, units	2018, ISTAT
Supermarkets, units	2018, ISTAT
Ipermarkets, units	2018, ISTAT
Airports, units	2018, ISTAT
Landed and departed passengers in airports, units	2018, ISTAT
Landed and departed airplanes in international flights, units	2018, ISTAT
Healthcare institutes (private and public), units	2018, Ministry of Health
Public healthcare institutes, units	2018, Ministry of Health
Days of stay in public and private healthcare institutes	2015, ISTAT
Days of stay in public healthcare institutes	2015, ISTAT
Patients in public and private institutes, units	2015, ISTAT
Patients in public institutes (except for residual psychiatric institutes), units	2015, ISTAT
Beds in pneumatology in public and private healthcare institutes	2015, ISTAT
Mechanical lung ventilators, units	2018, Ministry of Health
People with at least 1 chronic disease, units	2017, Ministry of Health
People with at least 2 chronic diseases, units	2017, Ministry of Health
% people with diabetes	2017, Ministry of Health
% people with hypertension	2017, Ministry of Health
% people with bronchitis	2017, Ministry of Health
% people with osteoporosis	2017, Ministry of Health
% people with arthritis	2017, Ministry of Health
% people with allergy	2017, Ministry of Health
% people with ulcer	2017, Ministry of Health
Old-age index	2016, ISTAT
Life expectancy at birth (female)	2017, ISTAT
Life expectancy at birth (male)	2017, ISTAT
Active buses per 1000 habitants	2016, ISTAT
% children going to school with public transportation	2016, ISTAT
Public expenditure in healthcare per capita	2016, ISTAT
Factor risk: alcohol	2016, ISTAT
Factor risk: smoke	2016, ISTAT
Factor risk: obesity	2016, ISTAT
Average household income	2015, ISTAT
Mobility index (commuting due to work)	2011, ISTAT
Self-containment index	2011, ISTAT
Public mobility index	2011, ISTAT
PM10	2017, ISTAT
PM2.5	2017, ISTAT
ICU beds per 100K inhabitants	2018, Ministry of Health
Beds in pneumatology	2018, Ministry of Health
Additional beds in ICU on April, 10th 2020	2020, DPC
Weighted PM10	2018, ISTAT
Average students per classroom	2018, Ministry of Education
Average students per school	2018, Ministry of Education
Gini index for schools	2018, Ministry of Education
Average beds per nursing home (ward)	2018, Ministry of Health
Average beds per nursing home (whole)	2018, Ministry of Health
Gini index for nursing homes	2018, Ministry of Health
Average beds per hospital (ward)	2018, Ministry of Health
Average beds per hospital (whole)	2018, Ministry of Health
Gini index for hospitals	2018, Ministry of Health
Average number of employees	2017, ISTAT
Gini index for firms	2017, ISTAT
Total number of tests between February 25th and April, 30th 2020	2020, DPC
Total number of tests between February 25th and March, 23rd 2020	2020, DPC
Total number of tests between March, 23rd and April, 30th 2020	2020, DPC
Adults per family doctor	2017, Ministry of Health
Average members per family	2018, ASR Lombardia
Public transport rides per capita	2017, ISTAT

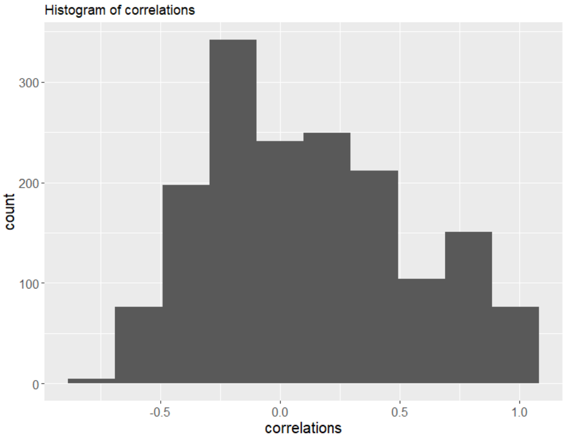

Figure S12. Correlations between all covariates. Histogram showing the distribution of pair-wise correlations for all the 68 covariates in Table S3. 24% of the correlations exceed 0.5 in absolute value. Note: Variance Inflation Factors cannot be computed for the whole set of 68 variables since the sample size here is only $n = 20$ (the Italian regions).

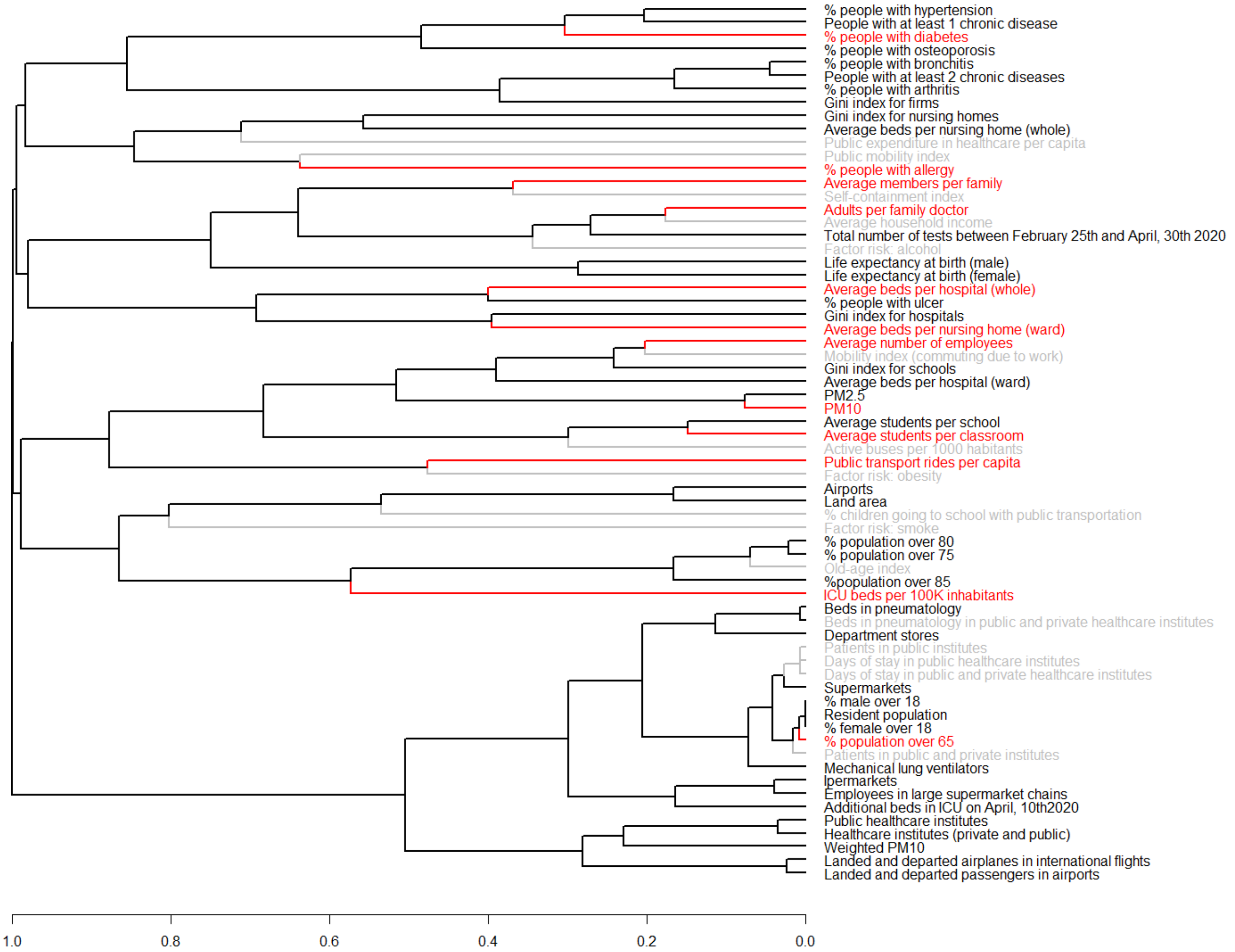

Figure S13. Correlation-based dendrogram for all covariates. Dendrogram of all the covariates in Table S3, based on the correlation distance $d(x_1, x_2) = 1 - |corr(x_1, x_2)|$ and complete linkage. The 12 selected covariates are shown in red, while covariates dating 2016 or earlier are in grey.

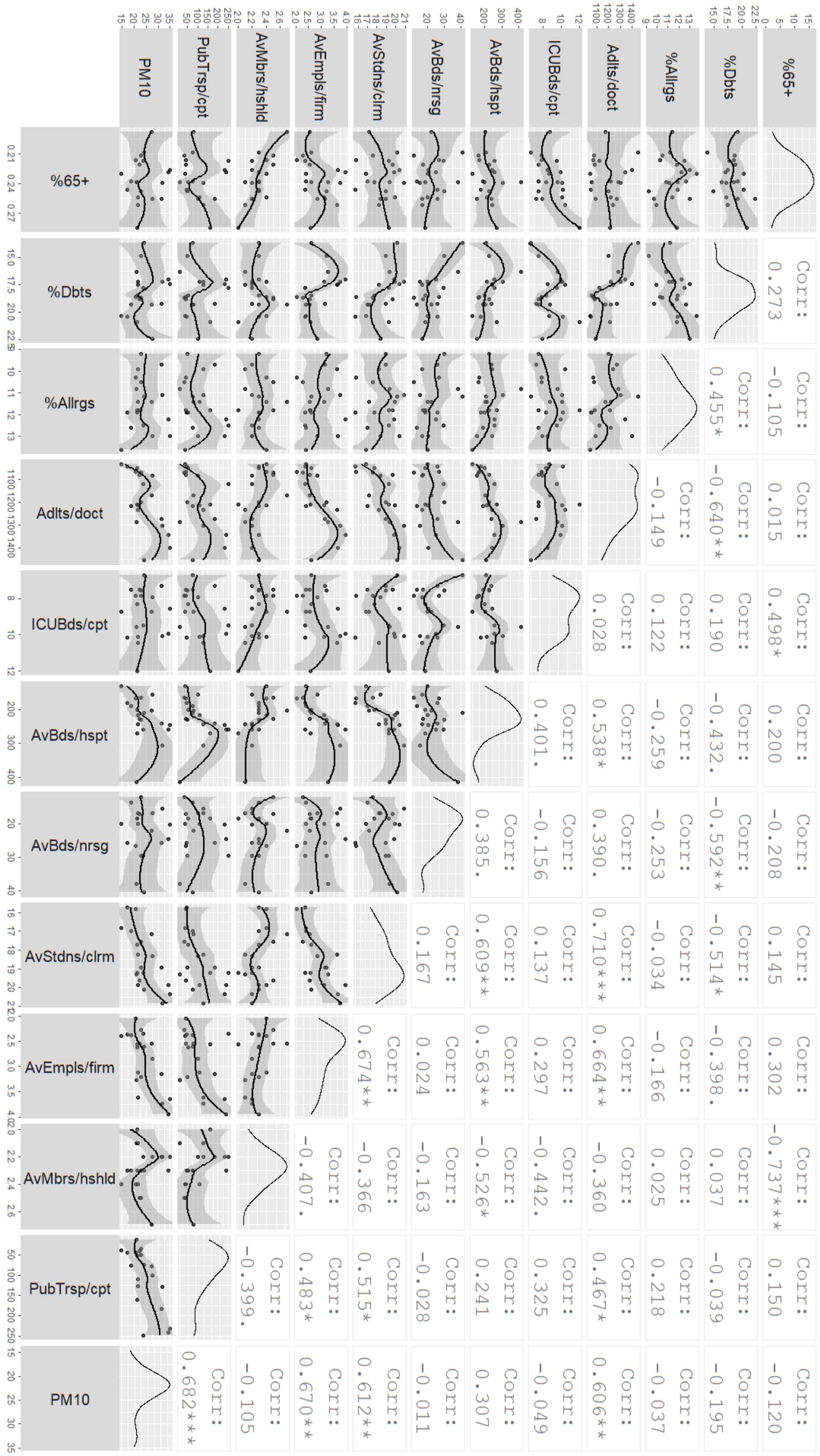

Figure S14. Associations between the 12 selected covariates. Exploratory matrix containing scatterplots with loess regressions, marginal densities, and correlation between pairs of covariates.

Table S4. Variance Inflation Factors of covariates. Variance Inflation Factors (VIF) for the 12 scalar covariates used in the main analysis.

Covariate	VIF
%65+	11.315684
%Dbts	12.105778
%Allrgs	3.933976
Adlts/doct	4.245479
ICUBds/cpt	2.892715
AvBds/hspt	5.012343
AvBds/nrsg	2.428207
AvStdns/clrm	6.529208
AvEmpls/firm	7.854636
AvMbrs/hshld	6.223056
PubTrsp/cpt	4.293915
PM10	12.858811

marginal $\beta(t)$ signs: 1 intercept model

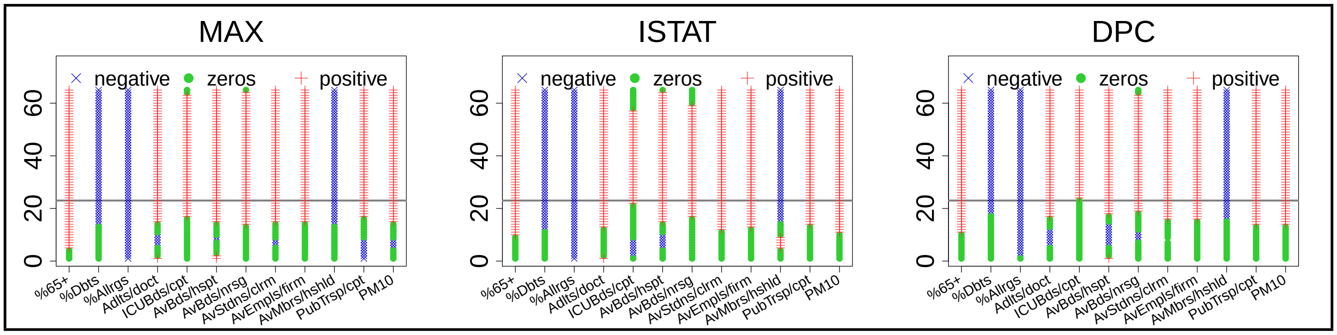

marginal $\beta(t)$ signs: 2 intercepts model

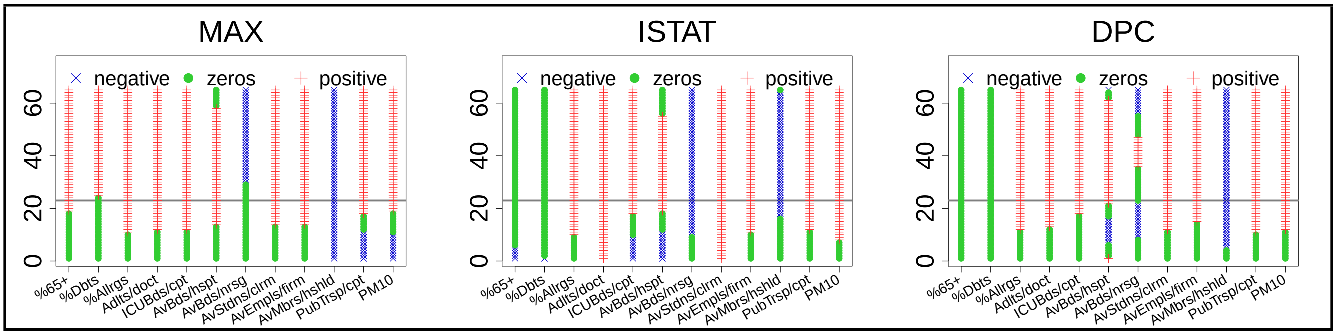

Figure S15. Marginal function-on-scalar regressions. Results for marginal function-on-scalar regressions. Mortality curves are regressed against each of the scalar covariates in Table 1. The top-row displays the signs of the effect curves estimated when just one intercept is included in the model. The bottom-row displays the signs of the effect curves estimated when we consider two different intercepts for curves in Group 1 and curves in Group 2. Time is on the vertical axis (the national lockdown on March 9, without shift, is marked by a horizontal line). Red, blue and green indicate, respectively, positive, negative, and non-significant portions (i.e., where 95% confidence bands around the estimated effect curve are entirely above, entirely below, or contain 0).

Table S5. Function-on-scalar feature selection. Top five scalar covariates selected by SsNAL-EN considering as response the MAX, ISTAT, and DPC mortality curves.

	MAX	ISTAT	DPC
1	Adults per family doctor	Adults per family doctor	Adults per family doctor
2	Ave. beds per hospital (whole)	Ave. students per classroom	Ave. beds per hospital (whole)
3	Ave. students per classroom	Ave. beds per hospital (whole)	Ave. students per classroom
4	Ave. members per household	Ave. employees per firm	Ave. employees per firm
5	Ave. employees per firm	Ave. members per household	Ave. members per household

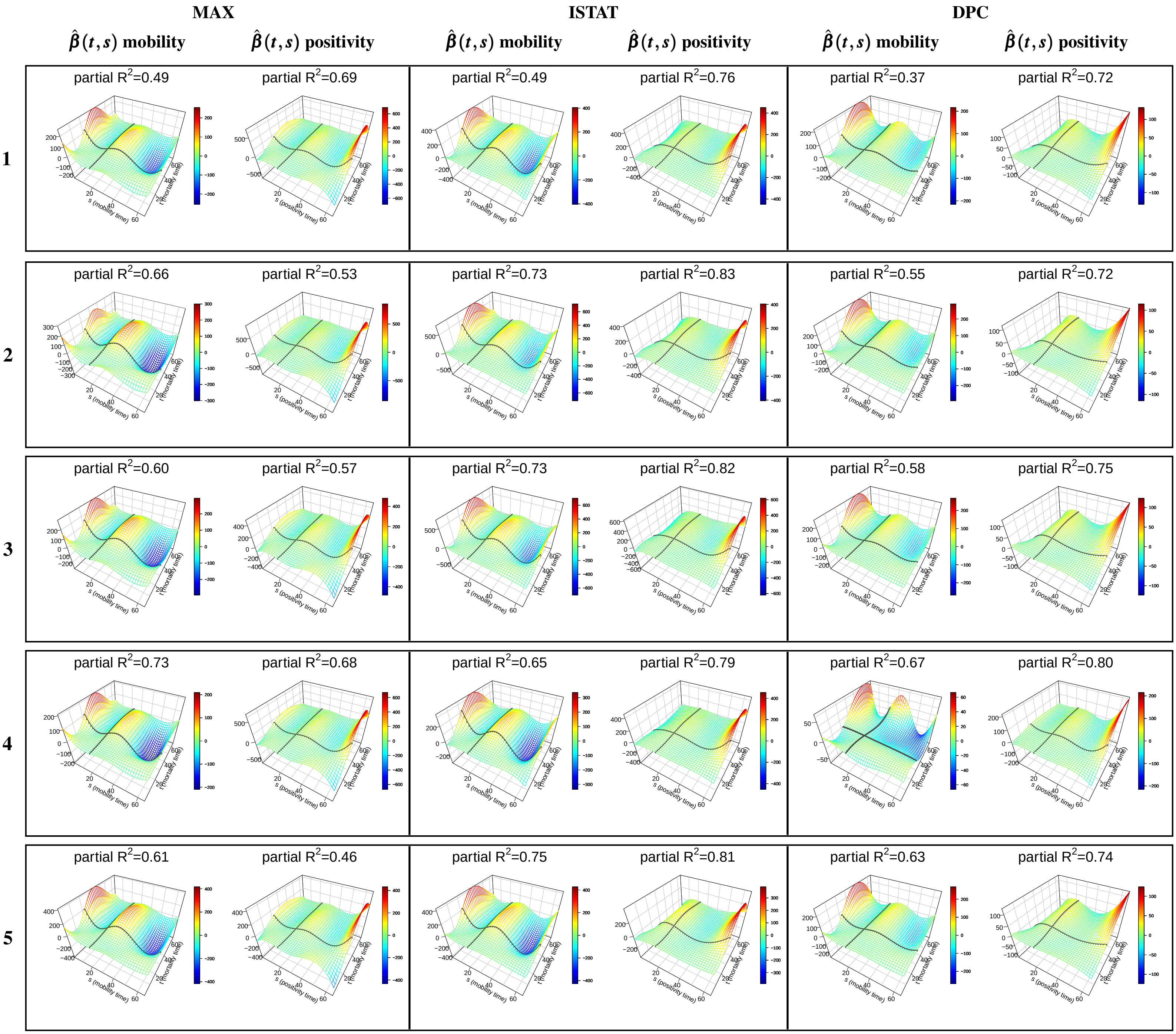

Figure S16. **Function-on-function regression of mortality on mobility, positivity, and a control scalar covariate.** Each row shows some results from the joint function-on-function regression mortality on local mobility, positivity, and one of the top 5 covariates selected by SsNAL-EN, used as control. In particular, we display the estimated effect surfaces for mobility and positivity (the March 9 date, without shift, is marked) with their respective partial R^2s. The scalar control covariates associated with each row are the following: 1: Adults per family doctor, 2: Ave. beds per hospital (whole), 3: Ave. students per classroom, 4: Ave. employees per firm, 5: Ave. members per household

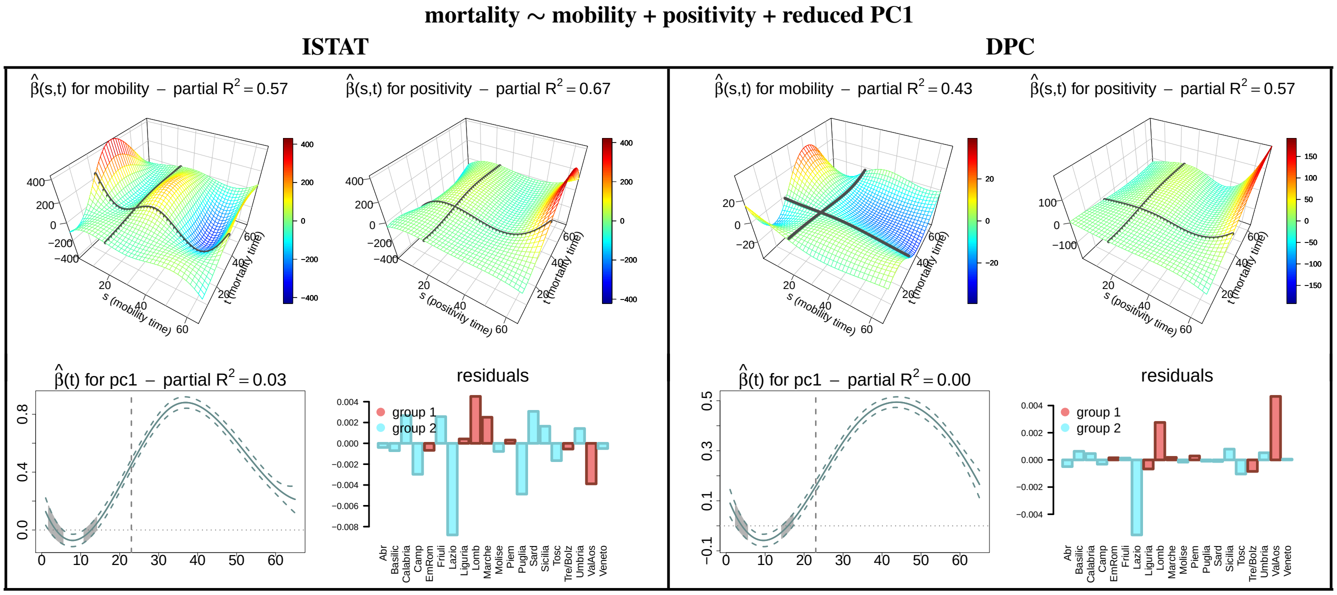

Figure S17. **Associating mortality to mobility, positivity and first principal component - ISTAT and DPC.** Results from the joint function-on-function regression of ISTAT and DPC mortality on mobility, positivity, and the first principal component (pc1) of the top 5 covariates, used as a "summary" control. Each panel shows the estimated effect surfaces for mobility and positivity and the estimated effect curve for pc1 with respective partial R^2 (for in-sample R^2 and LOO-CV R^2 see Table S2). For interpreting the regression residuals see Fig. S8.
